# Social relationships in adolescence and heavy episodic drinking from youth to midlife in Finland and Sweden — examining the role of individual, contextual and temporal factors

**DOI:** 10.1186/s12889-018-5885-8

**Published:** 2018-08-10

**Authors:** Noora Berg, Olli Kiviruusu, Christopher G. Bean, Taina Huurre, Tomi Lintonen, Anne Hammarström

**Affiliations:** 1 Department of Public Health and Caring Sciences, Uppsala University, PO BOX 564, 75122 Uppsala, Sweden; 2 Department of Public Health Solutions, National Institute for Health and Welfare, PO BOX 30, 00271 Helsinki, Finland; 3 Department of Health and Social Welfare, City of Vantaa, Vantaa, Finland; 4 Finnish Foundation for Alcohol Studies, c/o National Institute for Health and Welfare, PO BOX 30, 00271 Helsinki, Finland; 5 Faculty of Social Sciences, University of Tampere, Tampere, Finland

**Keywords:** Alcohol, Bioecological theory, Finland, Follow-up, Heavy episodic drinking, Life course, Social relationships, Sweden

## Abstract

**Background:**

Applying the Process-Person-Context-Time (PPCT) model of the bioecological theory, this study considers whether proximal processes between the individual and the microsystem (social relationships within family, peer group and school) during adolescence are associated with heavy episodic drinking (HED), from youth to midlife, and whether the macro level context (country) plays a role in these associations.

**Methods:**

Participants of two prospective cohort studies from Finland and Sweden, recruited in 1983/1981 at age 16 (*n* = 2194/1080), were followed-up until their forties using postal questionnaires. Logistic regression analysis was used to examine associations between social relationships at age 16 and HED (at least monthly intoxication or having six or more units of alcohol in one occasion) at ages 22/21, 32/30 and 42/43. Additive interactions between microsystem settings, as well as between settings and country, were also considered.

**Results:**

Consistent with the PPCT model, we found individual, contextual and temporal aspects to be associated with drinking habits. Higher levels of poor family relationships were associated with an increased likelihood of HED (ages 22/21 and 32/30) in both Finnish women and men and Swedish men. Higher levels of peer contact were associated with an increased likelihood of HED in both Finnish women (ages 32 and 42) and men (ages 22 and 32), and Swedish men (age 21). In contrast with the other groups, poorer relationships with classmates were associated with an increased likelihood of HED (age 30) for Swedish women only. For women, the combined effect of having both daily peer contact and living in Finland for HED at age 42/43 was statistically distinguishable from a pure additive effect.

**Conclusions:**

Micro and to a lesser extent macro level contexts are associated with heavy episodic drinking well into adulthood. The most relevant processes in the adolescent microsystem occur in family and peer settings. However, long-lasting protective or risk-raising effects between different settings and later HED were not found. Promoting good relationships across different contexts during adolescence may reduce the incidence of HED in adulthood.

## Background

Risky drinking habits, including heavy episodic drinking (HED), comprise a major burden for public health [1]. The definitions of HED vary, but it is usually defined as drinking 4–6+ alcohol units in one occasion. HED is associated with short and long term health problems, and social and socioeconomic harms to self, others and for society at large [1]. HED is common in Europe, but it usually decreases with age, notably after a person enters their thirties [2]. One of the challenges is to identify those who continue this detrimental health habit into later adulthood. Thorough understanding of the complex developmental processes related to alcohol use requires deeper knowledge on the individual, contextual and life course aspects, and especially their interrelations that influence this development [3]. Combining these aspects is not common in previous empirical studies on alcohol use. Bronfenbrenner’s bioecological theory of human development and the Process-Person-Context-Time (PPCT) model [4] provides a useful framework for understanding the complex relations between an individual, their environment, and the passage of time in influencing drinking habits.

### Process

In the PPCT model the first concept ‘process’ refers to dynamic interplay between the individual and their environment (including other persons, objects and symbols) through which development occurs. Proximal processes refer to associations between an individual and their immediate environment. This immediate environment has been referred to as the ‘microsystem’. Social relationships represent a domain where proximal processes occur between an individual and other persons. In this study we focus on processes occurring in adolescence; a time when interrelations between an individual and their family, peers, and school, constitute important processes that effect the person’s development. Adolescence is often a time of rapid changes in relationships; child-parent relationships are often renegotiated [5] and the importance of relationships in other growth milieus strengthen. For example, life style choices and decisions to engage in risky behaviours such as alcohol use may be more influenced by peer relationships [6]. However, despite the increasing influence of other factors, family relationships retain an important role in the lives of adolescents [5, 7]. Many studies have examined the effects of family and peer relationships on adolescent alcohol use. Several previous studies have concluded that good adolescent family relationships protect against adolescent heavy alcohol use [8–10]. However, a systematic review found mixed evidence for parent-child relationships and adolescent alcohol use, and called for more detailed and versatile information on alcohol use [11]. In addition, adolescents who are more peer-oriented tend to use more alcohol [12, 13], while poor school bonding and environment (e.g. a student’s low attachment to teachers, poor school satisfaction) also increase the risk for alcohol use and drunkenness [10, 14]. Although there is some lack of clarity in the association between adolescent social relationships and adolescent alcohol use, the evidence for these concurrent associations is stronger than that for associations that extend beyond adolescence. Regarding associations between adolescent social relationships and later adulthood alcohol use the evidence is mixed. Some studies have found associations between poor family relationships (e.g. less harmonious relationships, family conflict) in adolescence and harmful alcohol use in later adulthood [15, 16], while others have not [17, 18] or have only found associations in either gender or only at some ages [19–22]. Findings regarding peer relationships (e.g. low peer acceptance) are equally mixed [18–20, 23]. Evidence suggesting associations between adolescent school environment and adult alcohol use is scarce [21].

### Person

In Bronfenbrenner’s PPCT model the ‘person’ component refers to an individual’s characteristics (e.g. age, sex, heavy episodic drinking). These are the characteristics that a person brings with them to any social situation [4, 24]. Men tend to drink alcohol in larger amounts and more often than women [25]. Whether the influence of the risk factors on drinking also differs between sexes is not so evident. Sex differences related to the role of family and other social relations are especially under studied [26]. Sex could also be seen as a more contextual measure (i.e. gender), but in this study we follow Bronfenbrenner’s view of sex as an individual level measure.

### Context

In the PPCT model ‘context’ describes the environment of the person, whereas ‘process’ describes the way the person and context are interrelated. In the PPCT model the ‘context’ can be divided into four different systems. The microsystem refers to the immediate environment of a person. For example in adolescence, family, peer group, school and hobbies form important settings in the microsystem. The mesosystem refers to interrelations between different settings within the microsystem. It has been suggested that constellations of relationships in different settings may matter more than relationships in any specific setting; for example family and peer influences can both contradict and reinforce each other [6], such as where good social relationships in the family setting (e.g. good family cohesion) can buffer against negative peer context influences (e.g. friends drinking alcohol) on alcohol use [27, 28]. In previous studies on social relationships in various settings and alcohol use, it has been common to examine the effect of one setting at a time, rather than the interplay between different settings. One study that did examine several different settings simultaneously found family setting to be a more important predictor of HED at age 33 than risky peer setting or school setting [21]. The exosystem refers to an outer layer of the context, not where the individual of interest is directly situated, but which has an indirect influence on their development (e.g. parent’s or spouse’s workplace). Finally, the macrosystem is the outermost layer, which refers to the overarching pattern of characteristics of a group, whose members share resources, hazards, lifestyles, opportunity structures, life course options and patterns of social interchange [24, 29] (e.g. government policies, economic circumstances, cultural expectations). In this study we consider the macrosystem in the form of two different countries: Finland and Sweden. While both countries are Nordic welfare states, some differences have occurred especially regarding differences in alcohol policies and drinking habits. HED is a common drinking habit in both countries, although more so in Finland than Sweden [1]. Alcohol use has increased in both countries from the 1960s onwards until very recently, but more rapidly in Finland [30]. Alcohol policies (regulation of sales, alcohol taxation, retail policy etc.) have been rather strict in both countries. Some macro level differences have also been observed regarding family and school. Regarding family policies Sweden has been a forerunner in introducing parental leave [31], and the culture and policies in general have promoted more equality between women and men than in Finland. No major differences in child-rearing practices have been observed between the countries, but it has been stated that based on “equality ideology”, even young children are treated as equal partners to other family members in Sweden [32] and in comparison, the Finnish interactional structure is less equal [33]. School systems in Finland and Sweden both strived for social equality in the 1980s, but the focus is now more on individuality [34].

### Time

The final concept ‘time’ adds temporal aspects to the model, which are central to the idea of development. Time can refer to the chronological age of an individual, but also to historical time. In this study, the focus is on the former in terms of individual development through different life stages. Adolescence is a crucial time regarding alcohol use, because it is often initiated during this time and the drinking habits adopted tend to persist into adulthood [35]. Although the associations between social relationships (i.e. social support, networks and integration) and health behaviours are well established, for a long time there has been a distinction between studies that focus on either adolescence or adulthood [6], with the life course perspective generally being overlooked. This point was noted in 2010 by Umberson et al. and studies have since examined associations between adolescent social relationships and later alcohol use, however few researchers have examined these issues with long follow-up times spanning over several life stages. One of the few studies examining the unique and combined effects of adolescent family, peer, and school settings on HED in adulthood found that family relationships were associated with HED at age 33 but not at age 21, concluding that some of the contextual risk factors may gain their salience as people age [21]. As such, it is important to study alcohol use in several different life stages.

Many previous studies applying the PPCT model have not considered all aspects of the model nor analyzed in detail the relations between those aspects [36]. The PPCT model emphasizes the importance of the interrelations between its four constituent concepts. For example, personal and contextual characteristics likely interact, but the details of these interactions may be blurred. It might be that on the macro level, the more drunkenness-oriented drinking culture in Finland is more detrimental to men, but how the supportive proximal processes may protect from this macro feature is unclear.

### Aims

Using Bronfenbrenner’s PPCT model as a framework, this study examines how adolescent proximal processes, namely social relationships in various settings and the interactions between them (mesosystem) are associated with heavy episodic drinking over the life course in women and men, and the role of the macrosystem (country) in these associations. The more specific study questions are as follows:

1. Are social relationships in various settings (family, peers, and school) at age 16 associated with heavy episodic drinking from youth to midlife in women and men?
2. Can relationships in one setting protect or strengthen the effects of poor relationships in other settings for the association with HED over the life course?
3. Does the macrosystem (i.e. country) have protective or risk-raising role on the effects of social relationships at age 16 on HED over the life course?

## Methods

### Populations

The data is sourced from two separate follow-up studies: The Stress, Development and Mental Health study (TAM) from Finland, and the Northern Swedish Cohort (NoSCo) from Sweden. The studies included all pupils who attended the last year of compulsory school at age 16 in Tampere, a city in southern Finland in 1983, or in Luleå, a town in the north of Sweden in 1981. At baseline (age 16) 2194 (96.7% of the target population) pupils in Tampere and 1080 (99.7% of the target population) pupils in Luleå completed questionnaires during school hours. The Finnish participants were followed-up using postal questionnaires at ages 22, 32 and 42. The Swedish participants were followed-up using survey questionnaires at class reunions and postal questionnaires at ages 21, 30 and 43 (Table 1.).

**Table 1:** Birth year, follow-up years and participation in the Finnish and the Swedish cohort

Cohort, Study population	Birth year	Adolescence	Youth	Early adulthood	Midlife
Finland *N* = 2269	1967	1983 / age 16 *N* = 2194	1989 / age 22 *N* = 1656 (75.5%)	1999 / age 32 *N* = 1471 (67.0%)	2009 / age 42 *N* = 1334 (60.8%)
Sweden *N* = 1083	1965	1981 / age 16 *N* = 1080	1986 / age 21 *N* = 1060 (97.8%)	1995 / age 30 *N* = 1046 (96.7%)	2008 / age 43 *N* = 1010 (93.3%)

As observed in Table 1, the attrition rate in the Swedish cohort has been extremely low, while somewhat higher in the Finnish sample. At the age 42 follow-up of the Finnish cohort, the non-participants were more frequently men and had poorer school performance at age 16 compared with the continuing participants. Non-participation at age 42 in the Finnish cohort was also associated with frequency of HED at age 16, but this difference can be explained by male preponderance among the non-participants.

### Measures

#### Social relationships at age 16 in family, peer group and school settings

For social relationship variables, indicators were selected that are most similar between the two cohorts (scales were from 1 to 5, if not otherwise stated). Transient conflicts with family during adolescence can be common, so using various measures of family relationships provides a more stable assessment of family relationships [37]. Proximal processes related to family setting consisted of four different indicators: 1) poor relationship with mother, 2) poor relationship with father, 3) poor general family atmosphere, and 4) not spending much time with family. In the Finnish cohort, the mother and father relationship variables (sum scales, range 3–15) were formed by three statements for each parent: i. ‘my mother/father is close to me’ (reversed), ii. ‘I often argue with my mother/father’, and iii. ‘I feel my mother/father understands me’ (reversed) (all statements: ‘totally disagree’ to ‘totally agree’). In the Swedish cohort, mother and father relationships were measured as one question each: ‘how is your contact with your mother/father?’ (‘very good’ to ‘very poor/no contact’). General family atmosphere was measured with response to one statement/question: ‘I feel the atmosphere at home is good’ (reversed) (‘totally disagree’ to ‘totally agree’) (Finnish cohort) / ‘how do you enjoy/like being at home?’ (‘very well’ to ‘very poorly’) (Swedish cohort). Time spent with family was also measured with response to one statement/question: ‘I spend most of my spare time with my family’ (reversed) (‘totally disagree’ to ‘totally agree’) (Finnish cohort) / ‘are you most often at home or away in the evenings?’ (‘almost always at home’ to ‘almost always away’) (Swedish cohort). The mean of these four family indicators was calculated to form a general poor family relationship index.

Relationships with peers and school settings were each assessed with a single question. Peer contact was measured with a question regarding time spent with friends: ‘how often do you spend time with friends outside school hours?’ (‘daily’ to ‘seldom’) (Finnish cohort) / ‘are you most often by yourself or with friends on spare time?’ (‘almost always with friends’ to ‘almost always alone’) (Swedish cohort). A lot of time spent with peers was classified as daily peer contact. Relations with classmates were assessed with a question: ‘how do you get along with your classmates?’ (4-point scale, ‘get along with everybody’ to ‘nobody’) (Finnish cohort) / ‘do you like being with your classmates?’ (‘very well’ to ‘very poorly’) (Swedish cohort).

All social relationship variables (if not originally) were recoded to follow a scale from 1 (best/lowest risk) to 5 (worst/highest risk). For the interaction analyses, the variables were dichotomized as close to the upper quartile (75%) as possible.

#### Heavy episodic drinking

The United Nations define HED as having at least 60 g of pure alcohol (5 units in Finland and Sweden) on at least one occasion in the past 30 days [38] and this definition was applied as accurately as possible in the present study. HED was dichotomized (yes/no). In the Finnish cohort at age 22, the participants were defined as ‘heavy episodic drinkers’ if they reported heavy drunkenness at least monthly and ‘not heavy episodic drinkers’ if they reported heavy drunkenness less often (on a scale of ‘once a week or more often’, ‛about 1–2 times a month’, ‛more seldom’ or ‘never’). At ages 32 and 42, HED was measured with a question ‘how often do you have six or more drinks in a row?’ from the Alcohol Use Disorders Identification Test (AUDIT) (‘never’, ‘less than monthly’, ‛monthly’, ‘weekly’ and ‘daily or almost daily’) [39]. Those who reported having six or more drinks in a row at least monthly were classified as ‘heavy episodic drinkers’, while those drinking less often (or not at all) were classified as ‘not heavy episodic drinkers’.

In the Swedish cohort the respondents reported the frequency of drinking occasions per beverage type (‘every or every other day’, ‘1-2 times a week’, ‘a couple of times a month’, ‘more seldom’ and ‘I do not drink this type of beverage’) and average intake of beer (number of bottles), wine (number of glasses), and strong beverages such as spirits (number of drinks) on a typical occasion. Respondents who reported drinking on average ≥ 5 bottles of beer, ≥ 5 glasses of wine, or half a bottle (37 cl) or more of strong beverages in one session and drinking at least a couple of times monthly were classified as ‘heavy episodic drinkers’. Respondents who reported drinking the aforementioned amounts more seldom (or not at all) were classified as ‘not heavy episodic drinkers’.

#### Country

Country variable was indicated by the cohort source i.e. Finland (TAM) or Sweden (NoSCo).

#### Control variables

In the Finnish study, HED at age 16 (baseline) was defined as being drunk at least four times during the school term (on average once a month) and not HED if drunkenness occurred 0–3 times. In the Swedish study, HED at age 16 was measured as at the other ages. For both cohorts, parental socioeconomic position (SEP) at age 16 was classified as ‘non-manual’ vs. ‘manual’ employment based on father’s occupation [40, 41].

### Statistical analyses

Bivariate and multiple logistic regression analyses with odds ratios (ORs) and 95% confidence intervals (CI) were used to examine how social relationships at age 16 are associated with HED at ages 22/21, 32/30 and 42/43. Analyses were performed for each microsystem setting (family, peer group, school) separately first with no adjustments (Model 1), and second controlling for HED and parental SEP at age 16 (Model 2). These analyses were stratified by country and sex.

The cohorts were then pooled and interactions between the three settings in adolescence (family, peer group, and school) as well as the macrosystem (Finland vs. Sweden) in relation to associations with HED were assessed with additive interaction analyses in multiple logistic regression models. The additive interaction analyses were performed with six combined variables (family/peers, family/school, peers/school, family/country, peers/country, school/country) (see classification in Table 4). Relative excess risk due to interaction (RERI) is a measure of additive interaction [42]. RERI = 0 indicates the absence of an interaction or mere additivity; RERI > 0 indicates a positive interaction or an effect that is more than additivity (i.e. having the two risk factors constitutes a more pronounced risk for an outcome than what would have been expected by just summing the separate risks of the two factors), while RERI < 0 indicates a negative interaction or less than additivity. The 95% CIs for the RERIs were calculated as suggested by Knol and VanderWeele [43]. An interval not including 0 indicates statistical significance. All analyses were made separately for women and men. IBM SPSS Statistics 22 [44] was used for data analyses.

## Results

Characteristics of the participants are summarised in Table 2. Men tended to be heavy episodic drinkers more often than women in both countries and Swedish women had a lower level of HED also compared to Finnish women, e.g. HED being twice as likely in Finnish women in the two last follow-ups. The logistic regression analyses for the HED outcomes for women and men, in Finland and Sweden are presented in Table 3.

**Table 2:** Characteristics of the participants

	Scale 1–5								Dichotomized^a^							
	Finland				Sweden				Finland				Sweden			
	Women *N* = 1071		Men *N* = 1123		Women *N* = 497		Men *N* = 545		Women *N* = 1071		Men *N* = 1123		Women *N* = 497		Men *N* = 545	
	Mean	(sd)	Mean	(sd)	Mean	(sd)	Mean	(sd)	%	(n)	%	(n)	%	(n)	%	(n)
Family																
Poor mother relationship^a^	2.1	(0.9)	2.0	(0.8)	1.5	(0.7)	1.4	(0.6)								
Poor father^a^ relationship	2.2	(0.9)	2.0	(0.8)	2.0	(1.1)	1.7	(1.0)								
Poor home atmosphere	2.0	(1.1)	1.8	(1.0)	1.6	(0.8)	1.5	(0.7)								
Does not spend most spare time with family/at home	3.6	(1.2)	3.6	(1.1)	2.8	(1.0)	2.9	(1.1)								
Poor family relationship index	2.5	(0.7)	2.4	(0.7)	2.0	(0.7)	1.9	(0.6)	32.2	(344)	23.9	(266)	26.6	(128)	20.0	(105)
Peers																
Daily peer contact	3.9	(1.2)	4.2	(1.1)	3.8	(1.0)	3.9	(1.0)	40.2	(430)	54.0	(604)	24.6	(118)	32.3	(169)
School																
Poor relationships with classmates	2.0	(0.8)	1.8	(0.8)	1.9	(0.8)	1.8	(0.7)	7.3	(78)	5.5	(62)	17.7	(85)	14.3	(75)
Heavy episodic drinking (HED)																
HED age 16									20.1	(214)	23.5	(262)	13.5	(65)	17.3	(91)
HED age 22/21									13.1	(116)	29.6	(226)	10.6	(51)	43.5	(229)
HED age 32/30									19.0	(146)	52.1	(338)	11.2	(54)	29.5	(155)
HED age 42/43									19.8	(133)	47.9	(267)	7.9	(38)	20.7	(106)
Parental socioeconomic position age 16																
Manual workers									50.5	(531)	49.7	(543)	48.8	(234)	51.7	(271)

^a^Regarding social relationships at age 16, cut off as close to 75% as possible

**Table 3:** Univariate and multiple logistic regression analyses of the associations between adolescent social relationships and heavy episodic drinking (HED)

	Finland						Sweden					
	HED 22		HED 32		HED 42		HED 21		HED 30		HED 43	
Women	*N* = 884		*N* = 770		*N* = 672		*N* = 481		*N* = 481		*N* = 482	
	OR	95% CI	OR	95% CI	OR	95% CI	OR	95% CI	OR	95% CI	OR	95% CI
MODEL 1												
Poor family relationship index^a^	**2.27**	**1.75–2.94**	**1.64**	**1.29–2.08**	**1.59**	**1.23–2.05**	**1.57**	**1.04–2.38**	**1.56**	**1.04–2.35**	**1.68**	**1.04–2.72**
Frequent peer contact	**1.21**	**1.01–1.45**	**1.34**	**1.12–1.60**	**1.49**	**1.22–1.81**	1.00	0.74–1.34	1.08	0.80–1.45	1.11	0.77–1.59
Poor relationship with classmates	1.10	0.86–1.42	1.06	0.84–1.34	0.83	0.65–1.07	**1.44**	**1.06–1.97**	**1.47**	**1.08–1.99**	**1.51**	**1.06–2.16**
MODEL 2												
Poor family relationship index	**1.95**	**1.48–2.57**	**1.46**	**1.13–1.89**	1.28	0.97–1.69	1.29	0.82–2.02	1.36	0.88–2.11	1.30	0.77–2.20
Frequent peer contact	1.07	0.88–1.29	**1.23**	**1.02–1.48**	**1.37**	**1.12–1.68**	0.88	0.64–1.20	0.99	0.73–1.35	0.95	0.65–1.39
Poor relationship with classmates	1.13	0.87–1.47	1.08	0.85–1.38	0.81	0.63–1.06	1.35	0.97–1.86	**1.40**	**1.02–1.92**	1.38	0.95–2.01
Men	*N* = 764		*N* = 649		*N* = 557		*N* = 526		*N* = 525		*N* = 511	
	OR	95% CI	OR	95% CI	OR	95% CI	OR	95% CI	OR	95% CI	OR	95% CI
MODEL 1												
Poor family relationship index	**2.29**	**1.78–2.94**	**1.61**	**1.26–2.06**	**1.36**	**1.05–1.76**	**2.77**	**2.02–3.80**	**1.91**	**1.42–2.58**	**1.47**	**1.06–2.05**
Frequent peer contact	**1.51**	**1.26–1.80**	**1.24**	**1.07–1.44**	1.13	0.96–1.34	**1.50**	**1.24–1.81**	1.16	0.96–1.42	1.16	0.92–1.46
Poor relationship with classmates	1.19	0.97–1.45	0.95	0.78–1.15	1.07	0.86–1.32	**1.34**	**1.06–1.70**	1.24	0.97–1.60	**1.43**	**1.08–1.90**
MODEL 2												
Poor family relationship index	**1.99**	**1.53–2.58**	**1.53**	**1.18–1.97**	1.24	0.95–1.62	**2.07**	**1.48–2.90**	**1.69**	**1.22–2.36**	1.18	0.80–1.72
Frequent peer contact	**1.37**	**1.14–1.65**	**1.19**	**1.02–1.38**	1.08	0.91–1.27	**1.41**	**1.16–1.71**	1.12	0.92–1.37	1.10	0.87–1.39
Poor relationship with classmates	1.16	0.95–1.43	0.95	0.78–1.16	1.02	0.82–1.27	1.22	0.94–1.58	1.17	0.91–1.51	1.32	0.99–1.77

Bolded are significant with 95% CI

Model 1: Unadjusted

Model 2: Adjusted for heavy episodic drinking (HED) and parental socioeconomic position at age 16

Variables in the left column are continuous

The number of cases (N) indicates those with information on the outcome, the actual N varies somewhat by examined predictor and model

For Finnish women in the unadjusted model, higher levels of poor family relationships and peer contact were associated with an increased likelihood of HED in all ages, while associations with poor relationships with classmates were not significant. After controlling for HED at age 16 and parental SEP, poorer family relationships were associated with an increased likelihood of HED at ages 22 and 32 in Finnish women. In addition, a higher level of peer contact was associated with an increased likelihood of HED at ages 32 and 42.

For Swedish women, higher levels of poor family relationships and poor relationships with classmates were consistently associated with an increased likelihood of HED at all ages in the unadjusted analyses, while only poorer relationships with classmates were associated with an increased likelihood of HED in the adjusted model and only at age 30.

In the unadjusted model for Finnish men, higher levels of poor relationships with family and peer contact were associated with an increased likelihood of HED at ages 22 and 32, but poor relationships with classmates were not. With regards to HED at age 42, only the association regarding poor family relationships was significant. After adjustments, the associations with HED at ages 22 and 32 remained, while the association with poorer family relationships and an increased likelihood of HED at age 42 became non-significant.

In the unadjusted model for Swedish men, all variables in adolescence were associated with an increased likelihood of HED at age 21. At age 30, only the association with higher levels of poor family relationships during adolescence remained significant. At age 43, both higher levels of poor family and classmate relationships, but not higher level of peer contact, were associated with an increased likelihood of HED. In the adjusted model, all associations except that for poor relationships with classmates remained significant at age 21. The association with poor family relationships remained at age 30, while none persisted at age 43.

**Fig. 1:**
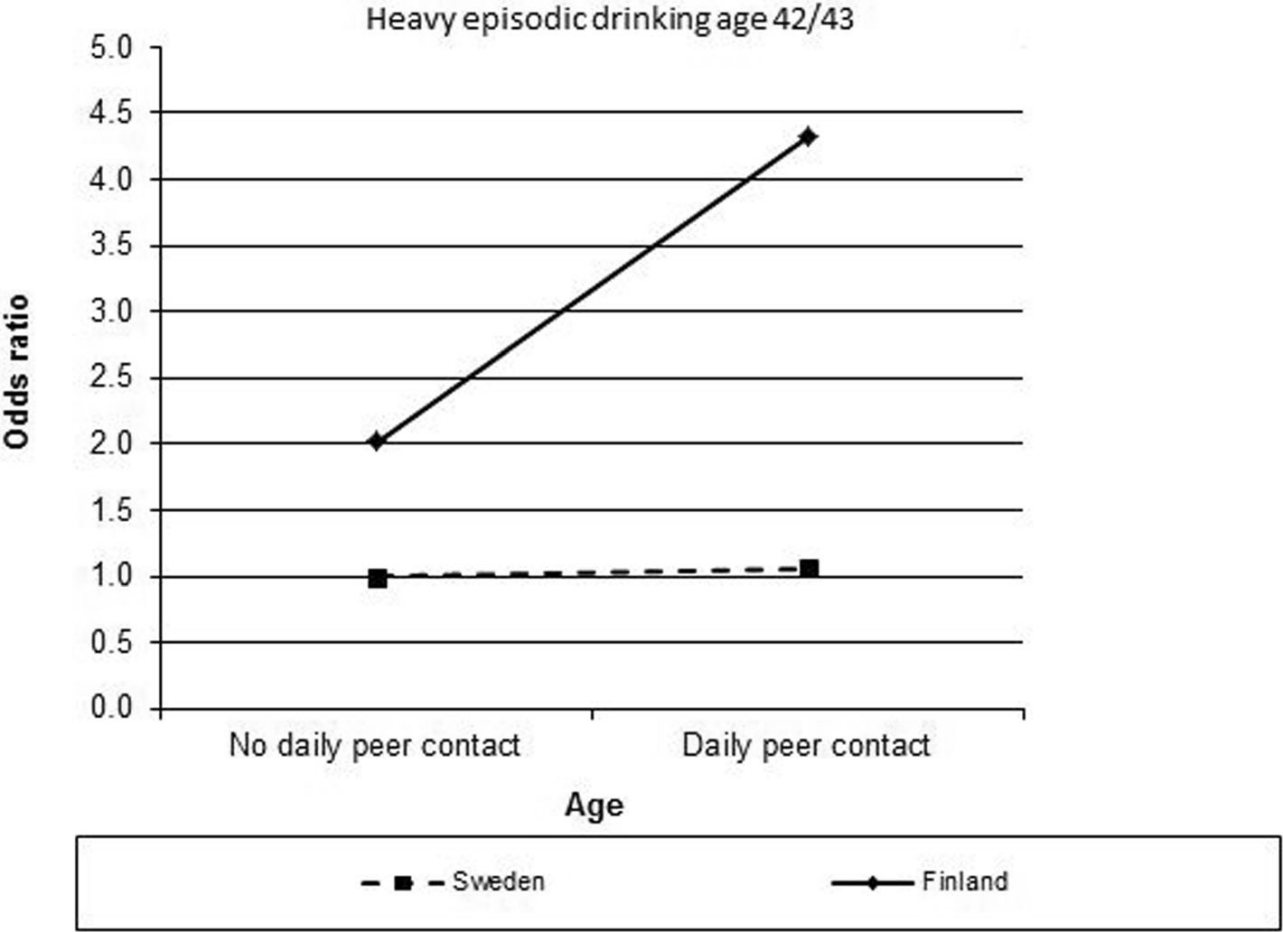
An additive interaction between peer contact and country for heavy episodic drinking (HED) at age 42/43 in women

Interactions were analysed using the concept of additive interactions in multiple logistic regression analyses (Table 4). RERIs with 95% CI were calculated for all outcomes. The results indicate a synergistic interaction between daily peer contact and country for HED at age 42/43 in women (Fig. 1), whereby those women who spent a lot of time with peers and were living in Finland had an increased risk for HED.

**Table 4:** Additive interaction analyses for women and men

	Women			Men		
	*N* = 1365	*N* = 1251	*N* = 1154	*N* = 1290	*N* = 1174	*N* = 1068
	HED 22/21	HED 32/30	HED 42/43	HED22/21	HED 32/30	HED 42/43
Good family-no daily peer	1	1	1	1	1	1
Good family- daily peer	0.82 (0.50–1.34)	1.36 (0.90–2.07)	**1.97 (1.24–3.11)**	**1.99 (1.48–2.66)**	1.26 (0.94–1.68)	1.15 (0.84–1.58)
Poor family-no daily peer	**1.72 (1.09–2.70)**	1.57 (1.00–2.45)	**1.91 (1.15–3.17)**	**2.19 (1.42–3.35)**	**1.97 (1.29–3.01)**	0.83 (0.49–1.39)
Poor family- daily peer	**1.87 (1.16–3.00)**	**2.11 (1.34–3.33)**	**2.81 (1.71–4.63)**	**3.34 (2.21–5.04)**	**2.36 (1.57–3.57)**	**1.59 (1.02–2.48)**
RERI (95% CI)	0.34 (− 0.66–1.33)	0.18 (− 0.89–1.25)	− 0.06 (− 1.57–1.45)	0.17 (− 1.32–1.65)	0.14 (− 1,01–1.30)	0.61 (− 0.11–1.33)
Good family-good school	**1**	**1**	**1**	**1**	**1**	1
Good family-poor school	1.36 (0.64–2.88)	0.40 (0.14–1.14)	0.28 (0.07–1.16)	1.02 (0.59–1.77)	1.35 (0.79–2.30)	1.12 (0.60–2.08)
Poor family-good school	**1.85 (1.27–2.70)**	**1.50 (1.05–2.14)**	**1.56 (1.06–2.29)**	**1.88 (1.36–2.58)**	**2.03 (1.47–2.81)**	1.09 (0.76–1.57)
Poor family-poor school	**2.70 (1.40–5.22)**	1.66 (0.84–3.28)	**2.29 (1.10–4.77)**	**2.61 (1.35–5.04)**	1.82 (0.97–3.39)	1.42 (0.71–2.85)
RERI (95% CI)	0.50 (− 1.48–2.47)	0.75 (− 0.47–1.98)	1.45 (− 0.26–3.16)	0.71 (− 1.12–2.54)	− 0.56 (− 1.98–0.86)	0.21 (− 1.01–1.42)
Good family- Sweden	1	1	1	1	1	1
Good family-Finland	1.08 (0.67–1.72)	**1.77 (1.14–2.73)**	**3.10 (1.82–5.27)**	**0.49 (0.37–0.65)**	**2.57 (1.93–3.41)**	**3.63 (2.64–5.00)**
Poor family-Sweden	1.73 (0.94–3.20)	1.69 (0.93–3.08)	**2.10 (1.03–4.28)**	**2.27 (1.41–3.65)**	**2.12 (1.34–3.34)**	1.26 (0.74–2.14)
Poor family-Finland	**2.16 (1.32–3.55)**	**2.77 (1.71–4.50)**	**5.12 (2.90–9.06)**	0.90 (0.61–1.33)	**4.76 (3.16–7.17)**	**3.92 (2.49–6.18)**
RERI (95% CI)	0.35 (− 0.77–1.48)	0.32 (− 0.95–1.58)	0.93 (− 1.22–3.08)	− 0.86 (− 1.94–0.22)	1.08 (− 0.78–2.93)	0.03 (− 1.70–1.75)
No daily peer-good school	1	1	1	**1**	**1**	1
No daily peer-poor school	1.49 (0.80–2.75)	0.80 (0.40–1.61)	1.37 (0.66–2.84)	1.63 (0.97–2.75)	1.38 (0.83–2.31)	1.32 (0.73–2.38)
Daily peer-good school	0.97 (0.67–1.42)	1.39 (0.99–1.95)	**1.99 (1.38–2.88)**	**2.05 (1.57–2.68)**	**1.34 (1.03–1.74)**	**1.35 (1.01–1.81)**
Daily peer-poor school	1.73 (0.75–3.98)	1.23 (0.49–3.08)	0.88 (0.25–3.06)	**2.21 (1.09–4.46)**	1.56 (0.78–3.12)	1.53 (0.71–3.30)
RERI (95%CI)	0.27 (− 1.38–1.91)	0.04 (− 1.24–1.32)	− 1.48 (− 3.12–0.15)	− 0.47 (− 2.22–1.28)	− 0.17 (− 1.44–1.11)	− 0.14 (− 1.52–1.25)
No daily peer- Sweden	1	1	1	1	1	1
No daily peer- Finland	0.96 (0.63–1.48)	1.42 (0.93–2.16)	**2.02 (1.22–3.33)**	**0.36 (0.25–0.51)**	**2.18 (1.58–3.02)**	**3.19 (2.20–4.61)**
Daily peer-Sweden	0.60 (0.29–1.27)	1.01 (0.52–1.94)	1.06 (0.48–2.30)	**1.61 (1.10–2.37)**	1.12 (0.75–1.68)	1.20 (0.76–1.91)
Daily peer-Finland	1.12 (0.71–1.76)	**2.24 (1.45–3.45)**	**4.32 (2.62–7.12)**	0.81 (0.60–1.11)	**3.12 (2.26–4.32)**	**4.42 (3.07–6.36)**
RERI (95% CI)	0.56 (−0.05–1.16)	0.81 (− 0.10–1.72)	**2.25 (0.65–3.85)**	−0.16 (− 0.76–0.44)	0.81 (− 0.20–1.84)	1.03 (− 0.37–2.43)
Good school-Sweden	1	1	1	**1**	1	1
Good school-Finland	1.22 (0.82–1.83)	**1.77 (1.22–2.56)**	**3.18 (2.03–5.00)**	**0.50 (0.38–0.64)**	**2.56 (1.97–3.32)**	**3.80 (2.82–5.13)**
Poor school-Sweden	1.50 (0.75–3.02)	0.99 (0.47–2.07)	1.47 (0.65–3.33)	1.30 (0.78–2.18)	1.33 (0.79–2.23)	1.50 (0.84–2.69)
Poor school-Finland	2.04 (0.94–4.44)	1.14 (0.45–2.85)	1.65 (0.54–5.07)	0.66 (0.32–1.36)	**3.29 (1.65–6.55)**	**3.17 (1.42–7.04)**
RERI (95% CI)	0.32 (− 1.42–2.05)	−0.61 (− 1.95–0.72)	− 2.00 (− 4.45–0.45)	−0.13 (− 0.94–0.67)	0.41 (− 1.89–2.70)	−1.14 (− 3.81–1.53)

Multiple logistic regression analyses for heavy episodic drinking at three life stages in relation to the combined variables family/peers, family/school, family/country, peers/school, peers/country school/country at age 16. Adjusted for heavy episodic drinking and parental socioeconomic position at age 16 and also for country in analyses of interaction between family/peer, family/school and peer/school. RERI with 95% CI for each heavy episodic drinking outcome. Bolded are significant with 95% CI

## Discussion

Applying bioecological theory and the PPCT model, this study examined adolescent social relationships as proximal processes in various contexts and their association with heavy episodic drinking from youth to midlife. We found poor adolescent family relationships to be associated with HED for participants during their twenties and thirties, but after taking into account HED and parental SEP at age 16, not once they reached their forties. Higher level of peer contact was also associated with later HED, while poor relationships with classmates were associated to a lesser extent. We did not find support for adolescent social relationships in one setting, neither family, peer group nor school, to be jointly associated with other settings on drinking behaviour in later life stages. Regarding the macro level, Swedish women were notably different from the Swedish men and Finnish men and women, demonstrating a lower level of HED, as well as a lack of associations between adolescent family and peer relations and subsequent drinking. This study combined contextual and life course perspectives, which has not been common in previous research on social relationships and alcohol use. Unlike most previous studies using Bronfenbrenner’s PPCT model, we applied all four concepts: Process, Person, Context and Time. In general, we found support for the relevance of all four concepts of this theoretical framework.

### Proximal processes

As adolescents make the transition to becoming independent, conflicts in the family may increase, especially if the process involves opposing parental control and trust, such as in the practice of engaging in alcohol use [45]. The direction of associations is likely complex; drinking may both increase family conflicts, while also being a way to relieve stress resulting from poor family relationships. The strongest evidence to support the association between poor parent-child relationships and subsequent alcohol use has been found in genetically informative multilevel studies on externalizing symptoms (e.g. substance use, conduct disorders) [46, 47]. The results of the present study support previous findings. Poor family relationships in adolescence were associated with later life HED in Finnish women and men, and Swedish men, after taking into account HED in adolescence. Nonetheless, this association was only relevant regarding more proximal life stages (i.e. ages 22/21 and 32/30, but not 42/43). Relationships with parents typically form the primary social environment and basic foundation for a person’s future development. Parents influence their children via numerous ways, such as their own alcohol use, attitudes, control, and support. Open and constructive communication about alcohol use is easier if the relationships between the adolescent and their parents are good [48].

In adolescence, peer relationships can have positive or negative effects on an adolescent’s behaviour; either by encouraging or protecting from risky behaviours, depending on the norms and values among peers. Spending a lot of time with peers during adolescence was associated with later HED in Finnish women (ages 32 and 42, [age 22 only in unadjusted model]) and men (ages 22 and 32), and in Swedish men (age 22) in the adjusted models. Although social contacts are generally good for adolescent development, it may present a risk for later drinking behaviour. This finding highlights the complexity of the role of adolescent social relationships in later drinking behaviour [26, 49]. Personality characteristics may in part explain this association, e.g. extroversion has been found to be associated with increased alcohol use/problems [50]. Unfortunately, we could not take into account whether the participants’ peers were heavy episodic drinkers, which would likely have shed more light on these findings. However, we have taken into account the person’s own HED, which usually correlates highly with peers’ drinking habits [27]. A British cohort study that examined many risk factors for alcohol problems found peer group deviance to be associated with alcohol problems at age 20 [16].

We found poor relationships with classmates to be a risk for later HED only for Swedish women (all ages), and after taking into account age 16 conditions, only at age 30. Previous studies have found school related factors to be associated with drinking. However, most of these studies have examined concurrent drinking during adolescence and it could be that the association regarding school does not persist to later life. In the transition from compulsory school (age 16) to further education the relationships with classmates often change. So perhaps the influence of these relationships is not as important as for those that exist outside school. Our results are in line with a study that examined adolescent family, peer and school settings and HED at ages 21 and 33 [21], which did not find school factors to predict subsequent HED. However, unlike some previous studies [10, 14], we were not able to measure the school environment more broadly (e.g. group characteristics), school or class level attitudes towards substance use, or a person’s interest in school, all of which have previously been found to be associated with concurrent alcohol use during adolescence. Our measure of school environment (relationships with classmates) is an indicator for sense of belonging in school and indicates peer relationships in one setting in life, but it does not necessarily tell the whole story if the most relevant peer relationships are outside one’s school class.

### Person

Sex differences in heavy episodic drinking were as expected; men tended to be heavy episodic drinkers more often than women in both countries. The Swedish women tended to drink less heavily than all three of the other groups. Later life HED was associated with poor family relationships and frequent peer contact for both Finnish and Swedish men even after adjustments. The associations among Finnish women resembled those found in men, but the same pattern was not observed among Swedish women. In contrast with the other groups, for Swedish women poor relationships with classmates was the only variable of interest associated with later life HED. This could be due to the low prevalence of heavy episodic drinkers among this group. The level of HED in Swedish women is likely actually lower than in other groups, but it may also be that Swedish women were more likely to mix different beverages and our measure does not capture their HED as effectively as with other groups. In the current study we followed Bronfenbrenner’s classification of sex as an indicator of an individual level characteristic. However, future studies with a focus on theory development could benefit from a broader view on gender indicating social order and contextuality, especially regarding studies on social relationships.

### Context

The results suggest that the most relevant settings in the microsystem are family and peer settings. The mesosystem refers to interactions between these settings; however we did not find any significant interactions between settings regarding subsequent HED. Previous studies have found a good family setting to protect from the detrimental effects of a risky peer setting on alcohol use [27, 28]. However, these studies have examined concurrent alcohol use in adolescence. In this study we did not find these kinds of protective elements regarding later drinking in adulthood, and it may be that this protective effect does not extend into later life stages.

The role of the macro level has been less studied regarding associations between social relationships and drinking. In this study we noted differences between the countries in the associations between adolescent social relationships and later life HED. In the additive interaction analyses, between country and both family relationships and peer contact, the odds ratios were generally higher for men in Finland compared to men in Sweden, regardless of the presence of the other risk factors.

In adjusted interaction analyses the only significant RERI was found between peer contact and country regarding HED at age 42/43 in women. It was observed that those exposed to both daily peer contact and a more drunkenness-oriented culture (Finland) had an increased risk of drinking heavily in midlife. Along with family, peers form a social context through which an adolescent is exposed to cultural norms and influences [27].

### Time

Most previous studies have examined the association between social relationships and concurrent alcohol use in adolescence or examined these associations in later life stages but using cross-sectional designs. This study demonstrates that these associations extend beyond adolescence. We found more associations between adolescent social relationships and more proximal HED (at age 22/21) than at later life stages, with the number of significant associations decreasing with age. This finding is consistent with the proximity hypothesis, which suggests that more proximal exposures (i.e. those occurring closer to the time of the outcome) are more important than distal exposures [51]. Conversely, the association between frequent peer contact and HED emerged only at ages 32 and 42 in Finnish women, which is consistent with an alternative hypothesis from Lee et al., which states that some effects become more pronounced as people age [21]. It is well known that late adolescence and youth are typically times of peak alcohol consumption, and after this HED usually decreases especially in women, possibly due to parenting responsibilities [52]. It may be that those women, who still drink heavily in their forties, deviate in other ways from those who follow the typical pattern and could also have other additional risk factors for drinking.

### Methodological considerations

The main strengths of this study are the long follow-up times and practically full participation rate at baseline in both cohorts. In the Swedish cohort the participation rate has continued to be extraordinarily high. Conversely, there was some attrition related to male sex and poor school performance at age 16 in the Finnish cohort’s follow-ups. Although there was attrition related to HED at age 16 in the Finnish cohort, it was explained by male preponderance among the non-participants at age 42, which suggests that the results were not highly biased regarding heavy episodic drinking.

Another particular advantage of this study is the possibility to examine the study questions with two separate but relatively similar datasets. While the measures used had been developed independently in the two cohorts they were harmonised as well as possible for the purposes of the present study. Still, the measures did differ between countries. The measure of HED in the Swedish cohort does not consider persons who combine different beverages as heavy episodic drinkers, if the amounts within one beverage type did not exceed the limit used in this study. Therefore the prevalence of HED in this cohort may be underestimated; yet the frequencies observed are rather similar to those found in other population based Swedish studies that measure HED more specifically [53]. In addition the measurement of HED changed in the Finnish study (i.e. ‘perceived drunkenness’ vs. ‘having ≥6 drinks in one occasion’). Perceived drunkenness has been shown to correlate with consumption of six units of alcohol in adolescence [54], suggesting that the HED measure generally captures the same drinking habit in all waves. Nonetheless, it should also be acknowledged that at age 22 the wording of the questionnaire in the Finnish cohort does refer to heavy drunkenness, so it may not capture milder drunkenness at that age, and this could explain the lower prevalence of HED in Finnish compared to Swedish men at this life stage (29.6 vs. 43.5%).

There are also some issues considering the measurement of social relationships that should be taken into account. The measures in different settings of the microsystem may have measured different aspects of social relationships. In family and school settings, the quality of the relationships was measured, while in the peer setting the time/frequency spent among peers was the focus.

The measurement of parental SEP in adolescence was based on the father’s occupation, and if not available on the mother’s. This decision to base SEP mainly on father’s occupation was made because of a rather large proportion of missing information on mother’s occupation in the Finnish cohort. It would be better to measure parental occupation based on both parents, but our procedure has been quite common in the 1980s.

We did not specifically examine the possible mechanisms behind the associations between adolescent social relationships and later HED [55]. For example, it may be that patterns and skills of social interaction are adopted in childhood and adolescence, which are further reflected in the social relationships experienced in adulthood. Difficulties in social relationships in adulthood could also be associated with concurrent drinking behaviours [6].

The participants lived in one Finnish or one Swedish city during adolescence. In Finland some regional differences in adolescents’ alcohol use have been found previously [56], and therefore the results are perhaps not generalizable to the whole Finnish population. However, the results are likely generalizable to Finnish urban adolescents of that time. The Swedish cohort has been shown to be comparable to the country as a whole with regard to socio-demographic and socio-economic factors as well as health status and health behaviors [57, 58].

Isolating the causes of historical change or different aspects of the macro level (e.g. drinking culture vs. alcohol policy) in alcohol use is very complex given the net of likely multiple influences that are present simultaneously. More detailed information on macro level differences and the distinction between age, historical period, and cohort effects, would benefit further studies to trace the phenomena in greater detail.

## Conclusions

Guided by bioecological theory and the Process-Person-Context-Time model, this study found proximal processes of poor adolescent family relationships and frequent peer contact to be associated with an increased likelihood of heavy episodic drinking well into adulthood. We did not find long-lasting protective or risk-raising effects between different settings (mesosystem). These results suggest the importance of early interventions and promotion of positive social relationships in adolescence. It is important to apply a holistic perspective such as the PPCT model, acknowledging the individual, contextual and temporal aspects when examining alcohol use and planning preventive actions and treatment. This requires further development of theories combining these perspectives, as well as new empirical data. Theoretical development is also needed in relation to sex/gender and incorporating wider aspects of gender order into the PPCT model.

## Abbreviations

HED: Heavy episodic drinking
NoSCo: Northern Swedish Cohort
PPCT: Process-Person-Context-Time model
SEP: Socioeconomic position
TAM: The Stress, Development and Mental Health study
